# Cas proteins: dodgy scaffolding in breast cancer

**DOI:** 10.1186/s13058-014-0443-5

**Published:** 2014-09-25

**Authors:** Giusy Tornillo, Paola Defilippi, Sara Cabodi

**Affiliations:** 1 Department of Molecular Biotechnology and Health Sciences, University of Torino, Torino, 10126 Italy; 2 European Cancer Stem Cell Research Institute and Cardiff School of Biosciences, Cardiff University, Cardiff, CF24 4HQ UK

## Abstract

The members of the Cas protein family (p130Cas/BCAR1, Nedd9/HEF1, EFS and CASS4) are scaffold proteins required for the assembly of signal transduction complexes in response to several stimuli, such as growth factors, hormones and extracellular matrix components. Given their ability to integrate and coordinate multiple signalling events, Cas proteins have emerged as crucial players in the control of mammary cell proliferation, survival and differentiation. More importantly, it has been found that alterations of their expression levels result in aberrant signalling cascades, which promote initiation and progression of breast cancer. Based on the increasing data from *in vitro*, mouse model and clinical studies, in this review we will focus on two Cas proteins, p130Cas/BCAR1 and Nedd9, and their coupled signalling pathways, to examine their role in mammary cell transformation and in the acquirement of invasiveness and drug resistance of breast cancer cells.

**Electronic supplementary material:**

The online version of this article (doi:10.1186/s13058-014-0443-5) contains supplementary material, which is available to authorized users.

## Introduction

Mammary gland development is the result of coordinated actions of hormones, growth factors and extracellular matrix signalling pathways. Not surprisingly, deregulation of proteins acting at various points within these pathways can contribute to the onset and progression of breast cancer. Cas proteins serve as scaffolds to regulate the assembly of intracellular signalling complexes downstream of numerous receptors, such as growth factor and hormone receptors as well as integrins. Thus, they play a key role in integrating multiple signalling networks, which together ultimately govern cell survival, proliferation and migration [1].

**Figure 1:**
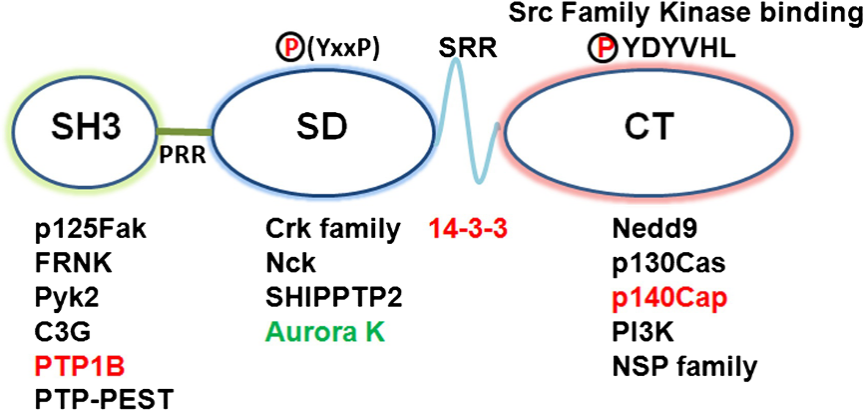
**p130Cas/BCAR1 and Nedd9 structural features and main interacting proteins.** Schematic representation of Cas protein main domains: an amino-terminal SH3 domain followed by a proline-rich region (PRR), a substrate domain (SD), a serine rich region (SRR), and a carboxy-terminal domain (CT). The main proteins whose interaction has been demonstrated for both p130Cas and Nedd9 are indicated in black, the interactors specific for p130Cas are in red and those specific for Nedd9 are in green. Noteworthy, p125FAK binds to the SH3 domain while the Src family kinases bind to the CT domain. The substrate domain with the multiple YxxP motifs represents the site where Src family kinases extensively phosphorylate Cas proteins.

The Cas family comprises four members: p130Cas/BCAR1 (p130 Crk-associated substrate; also known as Breast cancer anti-estrogen resistance 1 (BCAR1)), Nedd9 (Neural precursor cell expressed, developmentally down-regulated 9; also called Human enhancer of filamentation 1 (HEF-1) or Cas-L), EFS (Embryonal Fyn-associated substrate) and CASS4 (Cas scaffolding protein family member 4). These proteins have a similar structure, which is characterized by the presence of multiple protein interaction domains and several tyrosine and serine phosphorylation motifs [2] (Figure 1).

Over the past decade, p130Cas/BCAR1 and Nedd9 have emerged as key players in the control of many different aspects of mammary gland biology, including mammary epithelial cell homeostasis and mammary tumour cell behaviour. More interestingly, despite their high similarity, they regulate these processes through both distinct and overlapping mechanisms.

In this review, we summarize and discuss the most recent findings regarding the role of p130Cas/BCAR1 and Nedd9 in the normal mammary epithelium and in different breast cancer subtypes.

## p130Cas/BCAR1 and Nedd9 in the normal mammary epithelium

Several studies have shown that, in normal human breast tissue, both p130Cas/BCAR1 and Nedd9 are mainly present in the mammary epithelium rather than in stromal cells, although at relatively low levels and with a heterogeneous expression pattern [3]-[6]. Even if detected in all the distinct epithelial cell populations, p130Cas/BCAR1 expression is highly enriched in the basal compartment of the mouse mammary epithelium [4]. Similarly, Nedd9 is almost exclusively expressed in basal cells in the normal human mammary epithelium [7]. During late pregnancy and lactation p130Cas/BCAR1 is down-regulated [4], whereas the expression of Nedd9 has never been investigated at different stages of mammary gland development.

The regulation of p130Cas/BCAR1 levels is crucial for proper mammary morphogenesis and tissue homeostasis. Over-expression of p130Cas/BCAR1 enhances mammary branching morphogenesis *in vivo* during puberty as well as *ex vivo* upon stimulation with either epidermal growth factor or fibroblast growth factor [3],[8]. Moreover, in three-dimensional organotypic cultures of NmuMg mammary cells, high levels of p130Cas/BCAR1 elicit the formation of acini with a filled lumen [9]. Similar defects in lumen clearance can be evoked in primary mouse mammary explants when p130Cas/BCAR1 over-expression is specifically combined with epidermal growth factor and oestrogen treatment [8]. These indications for p130Cas having a role as a positive regulator of mammary cell growth are strengthened by the fact that *in vivo* over-expression of p130Cas/BCAR1 causes extensive hyperplasia throughout mammary gland development, delayed involution at the end of lactation, along with persistent proliferation, decreased apoptosis and hyper-activation of Src, Erk1/2 and Akt signalling pathways [3].

A likely explanation for the hyper-proliferative phenotype associated with high p130Cas/BCAR1 levels might be the expansion of the progenitor cell population that occurs following p130Cas/BCAR1 over-expression [4]. Up-regulation of p130Cas also impairs the ability of mammary luminal progenitors to differentiate in response to lactogenic stimuli and shifts their commitment towards the basal cell fate [4]. Remarkably, these alterations in the mammary progenitor cell behaviour depend on the abnormal activation of the c-Kit tyrosine kinase receptor, thus revealing a novel function of this receptor in the control of mammary cell differentiation [4].

The consequences of p130Cas/BCAR1 depletion *in vivo* in the mammary epithelium remain to be understood. Transgenic mice expressing the constitutively phosphorylated substrate domain (SD) of p130Cas/BCAR1 (MMTV- Src*/SD p130Cas), which has been reported to act as a p130Cas/BCAR1 dominant-negative mutant *in vitro*, do not exhibit any gross morphological alterations of the mammary gland [10]. However, the Src*/SD p130Cas mutant does not appear to effectively inhibit endogenous p130Cas/BCAR1 *in vivo*. Nevertheless, previous studies suggest that p130Cas/BCAR1 is required for mammary epithelial cells to survive and grow *in vitro*[9].

Lack of Nedd9 does not significantly affect mammary gland development [11], but whether p130Cas/BCAR1 might be able to compensate for Nedd9 deficiency remains to be investigated. Yet, more recently, it has been shown that in *NEDD9*-null mammary glands, the frequency of luminal progenitors is reduced, while the basal epithelial cell population is not altered [12]. However, although *NEDD9*-null mammary luminal progenitors show altered expression of genes involved in mitosis, replicative stress and centrosome maturation, no defect in their ability to grow *in vitro* has been observed. This finding, together with the selective expression of Nedd9 in basal cells reported by Bruna and colleagues [7], suggests that the effects of Nedd9 depletion in the luminal cell pool *in vivo* may be non-cell autonomous effects.

## p130Cas/BCAR1 and Nedd9 signalling in breast cancer: from cell transformation to tumour cell dissemination

The relevance of the adaptor proteins p130Cas/BCAR1 and Nedd9 in breast cancer has been highlighted by numerous *in vivo* and *in vitro* studies. Both p130Cas/BCAR1 and Nedd9 are abundantly expressed in human breast tumours and breast cancer cell lines [3],[5],[6],[13].

In particular, p130Cas/BCAR1 protein expression has been found markedly increased in a large subset of human breast tumours but with no correlation with tumour size or lymph node status. In spite of this, in patients with primary breast cancer, high BCAR1/p130Cas levels are associated with poor relapse-free survival and poor overall survival [13],[14]. Moreover, up-regulation of p130Cas/BCAR1 in benign breast lesions indicates that its over-expression may occur early during mammary tumourigenesis [3]. Like p130Cas/BCAR1, Nedd9 is strongly over-expressed in human breast cancers with respect to normal tissue. Specifically, Nedd9 expression positively correlates with a series of adverse prognostic markers, including high tumour grade [6] and metastatic disease [5],[15].

The mechanisms underlying the up-regulation of p130Cas/BCAR1 and Nedd9 in breast cancer are still largely unknown. However, as a concomitant increase of protein and mRNA levels has been reported [3],[6], alterations in their transcription and/or mRNA stability may contribute to deregulating the expression of these two genes in mammary tumours. To date, several signalling pathways relevant to breast cancer aetiology, including transforming growth factor (TGF)-beta, progesterone and hypoxia signalling, have been found to positively regulate Nedd9 mRNA expression [2], but less is known about the transcriptional control of p130Cas/BCAR1. In breast cancer cells, *BCAR1* transcription is directly activated by the early growth response protein 1 (EGR1) in response to phorbol esters, but the presence of putative binding sites for additional transcription factors, such as NF-kB, p53 and HIF, in the promoter region [16] points to a more complex control of *BCAR1* gene expression.

In the absence of an oncogenic hit, over-expression of either p130Cas/BCAR1 or Nedd9 is not sufficient to trigger mammary cell transformation. Nevertheless, up-regulation of these two Cas proteins seems to represent a convenient means for breast cancer cells to concomitantly boost multiple signalling pathways useful for tumour growth and invasion. Alternatively, increased Cas levels skew signalling cascades towards the preferential activation of specific downstream effectors.

For instance, high levels of p130Cas/BCAR1 in mammary epithelial cells subvert the function of TGF-beta from a negative regulator of tumour formation to a promoter of growth and dissemination. Elevated expression of p130Cas/BCAR1 diminishes the ability of TGF-beta to activate Smad2/3, but strengthens its coupling to p38 mitogen-activated protein kinase (p38 MAPK), thus rendering mammary cells resistant to TGF-beta-induced growth arrest. On the other hand, depletion of p130Cas/BCAR1 expression in transformed mammary cells increases the activation of Smad2/3 and suppresses their ability to invade in response to TGF-beta [9].

The involvement of p130Cas/BCAR1 in numerous pathways driving mammary cell transformation is due, at least in part, to its function as a mediator of integrin-dependent signalling. Adhesion to the extracellular matrix induces p130Cas/BCAR1 phosphorylation and association with Src and focal adhesion kinase (FAK), which in turn promote survival signalling and activation of the cellular migration machinery [1],[2].

Disruption of the interaction between p130Cas/BCAR1 and FAK is sufficient to inhibit mammary tumourigenesis driven by the polyoma middle T oncogene (PyMT) [17]. Moreover, activating mutations in the Ras or phosphoinositide 3-kinase (PI3K) pathway do not overcome the requirement of a functional p130Cas/FAK signalling complex for the survival and growth of human breast cancer cells [17]. A recent study shows not only that p130Cas/BCAR1 is necessary for Src-dependent mammary cell transformation but also that the interplay between p130Cas/BCAR1 and Src is strictly controlled by the E3 ubiquitin-ligase Cullin 5 (Cul5). Src-mediated phosphorylation of p130Cas/BCAR1 at specific tyrosine residues is responsible for Cas binding to the Cul5 adaptor SOCS6 and its consequent ubiquitylation by Cul5. Notably, loss of Cul5 results in p130Cas protein stabilization and facilitates the transformation of mammary epithelial cells by Src [18].

The ability of p130Cas/BCAR1 to control the remodelling of the actin cytoskeleton represents a mechanism commonly used to induce migration of breast cancer cells. For example, the enzyme lysyl oxidase (LOX) confers a motile phenotype on breast cancer cells by facilitating the formation of a p130Cas/Crk/DOCK180 complex, which sustains Rac activity and subsequent actin polymerization [19]. Conversely, the LOX pro-peptide (LOX-PP), which is released during the maturation of the pro-LOX enzyme, reduces breast cancer cell motility by attenuating phosphorylation and activation of p130Cas/BCAR1 [20].

Additional mechanisms that implicate p130Cas/BCAR1 in breast cancer invasion have been described. Tumour invasiveness often requires epithelial-mesenchymal transition, during which cells lose adhesions to their neighbours and become more motile. The link between p130Cas/BCAR1 and epithelial-mesenchymal transition is supported by the role of p130Cas/BCAR1 in the control of breast cancer cell plasticity. In fact, in the aggressive A17 mouse mammary tumour cells, p130Cas/BCAR1 silencing induces loss of mesenchymal features and acquirement of epithelial-like traits, including the re-expression of the cell-cell adhesion molecule E-cadherin. The mechanism whereby p130Cas/BCAR1 determines a mesenchymal and invasive phenotype resides in its capacity to positively regulate the expression of cyclooxygenase-2 in a Src- and Jnk-dependent manner [21]. Furthermore, in human mammary epithelial cells both p130Cas/BCAR1 and Nedd9 promote, via Src, removal of E-cadherin from the plasma membrane and its lysosomal degradation [22].

The first direct *in vivo* evidence for a role of Nedd9 in mammary tumourigenesis comes from the study of the effects of *NEDD9* gene ablation in the MMTV-PyMT mouse model [11]. This study revealed that depletion of Nedd9 impairs mammary tumour development by limiting the activation of multiple pro-oncogenic signalling proteins, including its binding partners Fak and Src as well as Ras downstream effectors. Specifically, due to a severe down-regulation of FAK activation, lack of Nedd9 results in defective cell spreading and migration and a greater susceptibility to anoikis [11].

Beyond its function as a mediator of a variety of integrin-dependent processes, such as cell adhesion, survival and migration, Nedd9 can further impact on mammary tumour cell behaviour through its ability to directly activate the mitotic regulatory kinase Aurora-A (AurA) [23]. In MCF-7 mammary tumour cells, up-regulation of Nedd9 results in enhanced AurA activity leading to centrosomal and mitotic spindle abnormalities, whilst a weaker effect can be seen after changes of p130Cas/BCAR1 expression [23].

**Figure 2:**
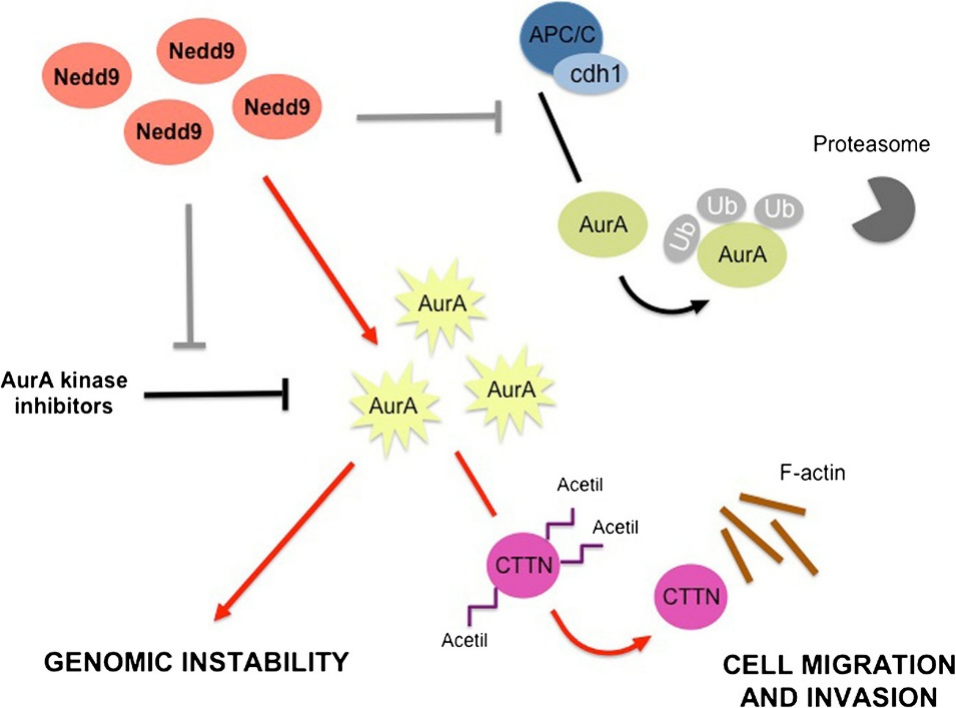
**Schematic representation of the mechanisms of Aurora-A kinase regulation by Nedd9 and major effects of Nedd9 up-regulation mediated by Aurora-A kinase in breast cancer cells.** AurA, Aurora-A; CTTN, cortactin; Ub, ubiquitin.

In human breast cancer AurA expression is considered an independent marker of poor prognosis [24] and it has been recently found that not only does it positively correlate with the expression of Nedd9 but also that co-expression of these two proteins has a significantly higher prognostic value [5]. Whilst most invasive mammary carcinomas highly express AurA, few have AurA gene amplification, implying that post-transcriptional mechanisms may be responsible for the alterations in AurA protein levels. Interestingly, binding of Nedd9 to AurA protects AurA from degradation by blocking its association with the APC/C ubiquitin ligase complex. As a consequence, Nedd9 overexpression in breast cancer cells leads to AurA protein stabilization and decreases the efficacy of AurA inhibitors [5] (Figure 2). In addition to its role in regulating the mitotic cell machinery, Nedd9 cooperates with AurA to control actin cytoskeleton dynamics and motility of breast cancer cells. Nedd9, through activation of AurA, negatively regulates cortactin (CTTN) acetylation and favours its binding to F-actin, thus sustaining cell migration and invasion [25] (Figure 2).

Increased levels of Nedd9 protein significantly correlate with the transition from *in situ* to invasive mammary carcinoma [15]. This observation is in line with a novel function ascribed to Nedd9 at the invasive pseudopodia of breast cancer cells. Nedd9 is able to direct local matrix degradation by reducing the levels of TIMP2 on the cell surface and favouring the activation of MMP14, MMP2 and MMP9 matrix metalloproteinases [15]. Nedd9 has also been identified as one of the key genes that mediate the switch from cohesive to single cell motility induced by TGF-beta during breast cancer cell dissemination. Up-regulation of Nedd9 by TGF-beta is indeed required for proper actin rearrangements at the leading edge and efficient amoeboid motility of migrating tumour cells [26]. Given the increasing evidence that depletion of Nedd9 drastically reduces migration and invasion of highly metastatic mammary tumour cells [5],[15],[25], inhibition of Nedd9 expression and/or activity represents an attractive strategy to target aggressive breast cancer cells. However, the complex scenario resulting from Nedd9 depletion should be taken into account. Indeed, silencing of Nedd9 can also cause centrosomal and spindle defects because of an abnormal AurA kinase activation [23]. As well, cells from MMTV-PyMT; *NEDD9*-null mammary tumours exhibit pronounced genomic instability, which ultimately facilitates the selection of cells with a more aggressive phenotype [27].

The contribution of p130Cas/BCAR1 and Nedd9 to specific oncogenic pathways in different breast cancer subtypes is described in detail in the paragraphs below.

## p130Cas/BCAR1 and Nedd9 in estrogen receptor-positive breast cancer

Expression of the oestrogen receptor (ER) in breast cancer inversely correlates with poor prognosis. Development and growth of ER-positive tumours are strictly dependent on the activity of ER, which, in addition to its well-described genomic action as a regulator of transcription, is also able to associate with cytoplasmic transducer molecules and activate non-genomic intracellular signalling cascades [28].

**Figure 3:**
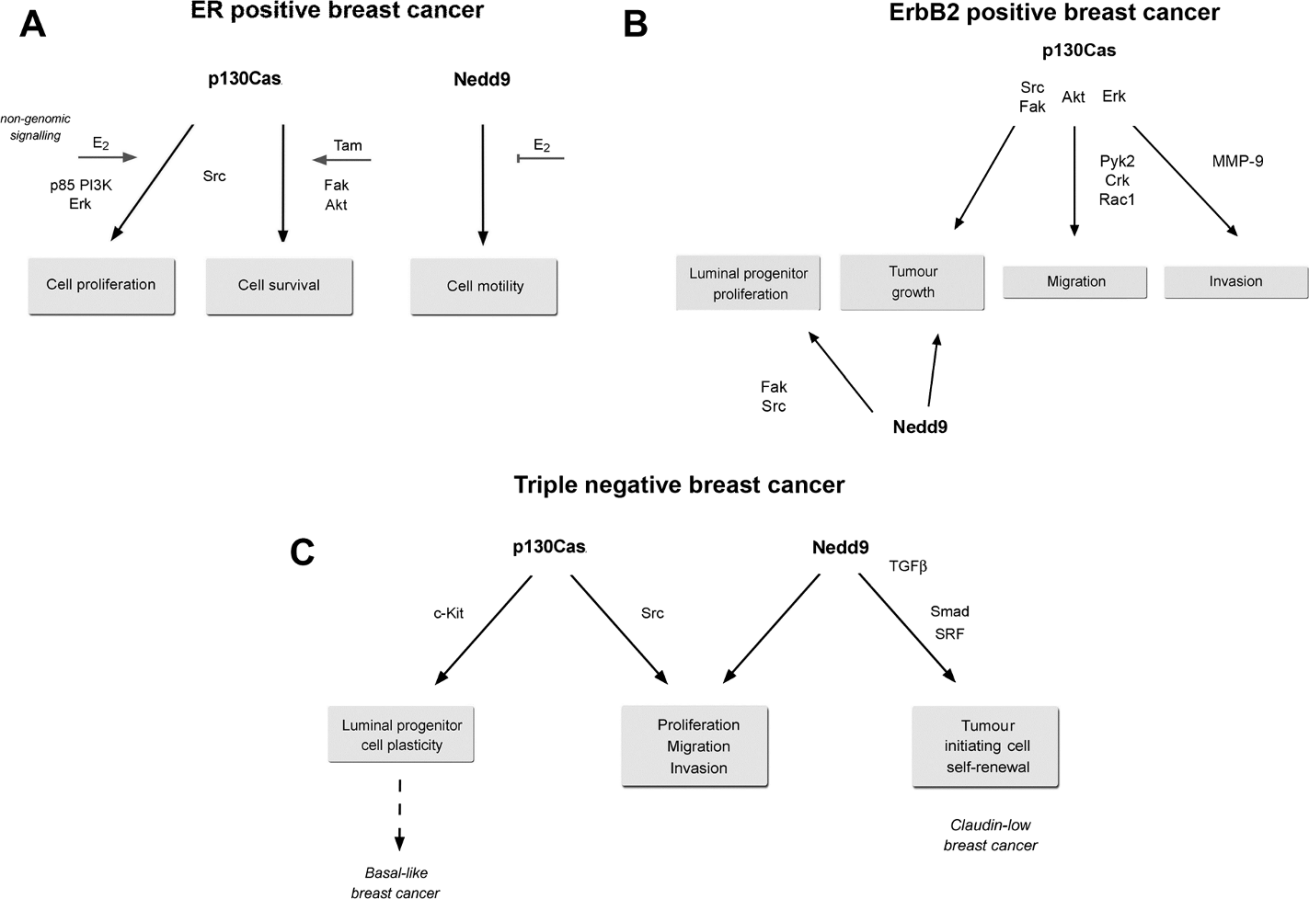
**Biological effects and signalling pathways involving p130Cas/BCAR1 and Nedd9 in different breast cancer subtypes. (A)** Estrogen receptor (ER)-positive breast cancer. **(B)** ErbB2-positive breast cancer. **(C)** Triple-negative breast cancer. PI3K, phosphoinositide 3-kinase; SRF, serum response factor; TGF, transforming growth factor.

In ER-positive breast cancer, high levels of p130Cas/BCAR1 are associated with poor prognosis and a higher risk of developing resistance to anti-oestrogen therapy [13],[29]. The increased aggressiveness observed for ER-positive breast cancer expressing high p130Cas/BCAR1 levels may arise from the p130Cas/BCAR1-dependent activation of non-genomic ER signalling. Indeed, high levels of p130Cas/BCAR1 in T47D breast cancer cells promote the assembly of a multi-protein complex including ERα, c-Src, and PI3K. In the presence of oestrogen, this macromolecular complex induces non-genomic ER proliferative signalling through Src and MAPK activation and the expression of cyclin D1. Conversely, silencing of endogenous p130Cas/BCAR1 is sufficient to markedly reduce oestrogen-dependent activation of Src and MAPKs as well as induction of cyclin D1 [30]. In parallel, in MCF-7 breast cancer cells, exposure to the selective ER modulator tamoxifen has been found to induce Src-mediated phosphorylation of p130Cas/BCAR1, which in turn promotes the activation of a pro-survival signalling pathway involving FAK and Akt [31] (Figure 3A).

At present, few data regarding a specific role for Nedd9 in ER-positive breast cancer are available, although, interestingly, its phosphorylation status has been linked to responsiveness to oestrogens. Indeed, ER-positive breast cancer cells preferentially express a hypophosphorylated form of Nedd9 (105 kDa Nedd9) rather than the highly phosphorylated form (115 kDa Nedd9), which is known to mediate cell spreading and migration [2]. In MCF7 cells, oestrogen treatment stabilizes the 105 kDa Nedd9 form and suppresses the ability of Nedd9 to induce cell spreading, thus suggesting that Nedd9 phosphorylation status might be an indicator of breast cancer cell motility in response to oestrogens [32]. However, the specific pathways that are activated following oestrogen-dependent changes of Nedd9 phosphorylation have not been defined yet (Figure 3A).

## p130Cas/BCAR1 and Nedd9 in ErbB2 breast cancer

Overexpression of the epidermal growth factor receptor 2 (ErbB2) occurs in approximately 25% of all breast cancers and is correlated with disease progression, decreased survival and metastasis. Such a poor prognosis is a likely reflection of the biological effects of ErbB2 overexpression, including increased cellular proliferation, anti-apoptosis, cell invasiveness and promotion of angiogenesis [33].

A positive correlation between the expression of BCAR1/p130Cas and ErbB2 has been found in human breast cancers and the co-expression of these two genes is associated with shorter overall survival and a higher risk of developing distant metastasis [34]. Moreover, concomitant expression of p130Cas and ErbB2 proteins is related to a higher proliferative index [3].

The role of p130Cas/BCAR1 as a mediator of ErbB2 signalling was initially demonstrated in breast cancer cell lines devoid of ErbB receptors, in which over-expression of ErbB2 was sufficient to induce cell migration by triggering the coupling between p130Cas/BCAR1 and Crk [35]. Further *in vivo* analyses have shown that in the MMTV-NeuT mouse model of ErbB2 tumourigenesis, over-expression of p130Cas/BCAR1 accelerates the onset of mammary tumours, which are characterized at the molecular level by increased activation of c-Src and Akt [3]. Consistently, silencing of p130Cas/BCAR1 is sufficient to impair growth of MMTV-NeuT spontaneous tumours [36]. Mechanistically, the synergistic action of p130Cas/BCAR1 and ErbB2 is due to the ability of p130Cas/BCAR1 to interact with Erb2 and mediate the assembly of a functional molecular complex consisting of ErbB2, c-Src, and FAK [36].

p130Cas/BCAR1 cooperates with ErbB2 not only in mammary cell transformation but also in driving breast cancer cell migration and invasion and formation of metastasis [34],[36],[37]. A recent *in vivo* study indicates the interaction between p130Cas/BCAR1 and the FAK-related kinase Pyk2 is required for ErbB2-transformed cells to invade and form metastasis [38]. Moreover, in MCF10A.B2 cells p130Cas/BCAR1 over-expression upon activation of ErbB2 induces cell invasion by strengthening ErbB2 downstream signalling and leading to enhanced Rac1 activation and MMP9 secretion [34],[36]. The invasive phenotype resulting from the concomitant p130Cas/BCAR1 over-expression and ErbB2 activation relies on a specific transcriptional program, which includes protein-coding genes and microRNAs involved in the control of amino acid synthesis, cell motility, migration, and angiogenesis [37] (Figure 3B).

The existence of mutual regulation between Nedd9 and ErbB2 is supported by independent studies showing that Nedd9 up-regulation increases the levels of ErbB2 mRNA [39] and, on the other hand, stimulation with the ErbB2 receptor ligand heregulin induces Nedd9 expression [40],[41]. The evidence of a functional role for Nedd9 in ErbB2-dependent tumourigenesis has been provided by the analysis of MMTV-ErbB2 mice with the genetic deletion of NEDD9, demonstrating that MMTV-ErbB2;Nedd9-null mice are strongly resistant to tumour formation. The requirement of Nedd9 expression at the early stages of ErbB2-dependent tumourigenesis reflects its role in the control of pre-tumourous luminal progenitor cell growth. Specifically, in the absence of Nedd9, MMTV-ErbB2-luminal progenitors exhibit profound defects in the activation of adhesion signalling pathways together with a markedly reduced proliferative potential [12].

Since loss of either Nedd9 or p130Cas/BCAR1 impairs growth of ErbB2 tumours, the full activation of crucial ErbB2 downstream effectors, such as Src and FAK, may require the co-expression of Nedd9 and p130Cas/BCAR1, suggesting that these two Cas proteins cooperate during early ErbB2 tumourigenesis (Figure 3B).

## p130Cas/BCAR1 and Nedd9 in triple-negative breast cancer

Triple-negative breast cancer (TNBC) is an aggressive breast cancer subtype defined by the lack of ER, progesterone receptor and human epidermal growth factor receptor 2 (ErbB2). Despite these distinctive histological features, TNBC comprises a highly heterogeneous group and encompasses a number of intrinsic molecular subtypes, most frequently basal-like and claudin-low [42].

Increasing data indicate that basal-like breast cancer originates from mammary luminal progenitors [43]. p130Cas/BCAR1 up-regulation is sufficient to confer basal cell features on luminal progenitors and to impair their full maturation in response to lactogenic stimuli. These defects in luminal progenitor differentiation together with the deregulation of the c-Kit receptor, which result from p130Cas/BCAR1 over-expression, closely resemble the phenotypes associated with Brca1 deficiency in the mammary epithelium [43]. Together, these observations indicate that p130Cas/BCAR1 up-regulation might be a priming event in the development of basal-like breast cancer. Consistent with this, p130Cas/BCAR1 has been found commonly over-expressed in human TNBC [4].

Phosphoproteomic data from human breast cancer cell lines have revealed that basal cancer cells exhibit higher tyrosine phosphorylation of p130Cas/BCAR1 compared with luminal cancer cells, despite similar p130Cas/BCAR1 expression in the two subgroups [44]. In line with this, tyrosine phosphorylation of the p130Cas/BCAR1 SD has emerged not only as a distinguishing feature of ER-negative breast cancer cell lines compared with ER-positive lines, but also crucial for the proliferation, migration and invasion of these cells [45] (Figure 3C).

Analysis of Nedd9 expression across different breast cancers has revealed a correlation of this gene with the TNBC subtype. TNBC cells require Nedd9 expression to maintain their mesenchymal phenotype and to migrate and invade *in vitro*[6]. Moreover, in contrast to ER-positive cells, ER-negative breast cancer cells predominantly express a hyper-phosphorylated form of Nedd9 (115 kDa Nedd9), which represents the pool of Nedd9 able to sustain cell adhesion and migration signalling [32].

Lastly, Nedd9 has been found over-expressed in claudin low breast cancers compared with other molecular subtypes. In claudin low breast cancer cell lines TGF-beta up-regulates Nedd9 expression by inducing Smad and MRTF-SRF protein binding to regulatory regions within the *NEDD9* locus. Nedd9 induction is necessary to establish a positive feedback loop that integrates TGF-beta/Smad and Rho-actin-SRF signalling and drives the expansion of tumour-initiating cells, selectively in claudin low breast cancer, upon TGF-beta stimulation [7] (Figure 3C).

## p130Cas/BCAR1 and Nedd9 in anti-oestrogen resistance

Tamoxifen is the most commonly used anti-oestrogen in the treatment of breast cancer. Patients with ER-positive breast tumours may initially benefit from this treatment, but a significant proportion of these initially responding patients acquire resistance to tamoxifen over time and the disease progresses [46].

p130Cas/BCAR1 has been identified as a gene responsible for the resistance to the anti-proliferative effects of tamoxifen [29],[47]. In general, in ER-positive breast cancer patients, high levels of p130Cas/BCAR1 correlate with earlier relapse and shorter overall survival [14].

A first hint as to the possible mechanisms underlying the up-regulation of p130Cas/BCAR1 in tamoxifen-resistant cells comes from the analysis of the *BCAR1* gene promoter. This study shows that *BCAR1* gene transcription is positively regulated by the transcription factor EGR1 and its co-regulator NAB2. Importantly, the increased expression of NAB2 and the enhanced EGR1 binding to the *BCAR1* promoter observed in tamoxifen-resistant cells is likely to be responsible for the constitutive high expression of p130Cas/BCAR1 [16].

Several mechanisms have been proposed to explain the correlation between p130Cas/BCAR1 expression and resistance to tamoxifen. A major determinant of the p130Cas/BCAR1-dependent increased proliferation of tamoxifen-resistant cells is the tyrosine phosphorylation of the p130Cas/BCAR1 SD. Indeed, tyrosine phosphorylation of the p130Cas/BCAR1 SD is induced by tamoxifen and correlates with tamoxifen-induced ER antagonist effects [31]. Either silencing of p130Cas/BCAR1 or abrogation of p130Cas/BCAR1 signalling, by expressing a dominant-negative tyrosine phosphorylated SD, is sufficient to re-establish sensitivity to tamoxifen in resistant cells. Inhibition of p130Cas attenuates the activation of Erk1/2 and PI3K/Akt pathways and results in cell death in response to tamoxifen [48].

Resistance to anti-oestrogen therapy specifically depends on the substrate domain of p130Cas/BCAR1 and not of Nedd9. Indeed, studies performed using chimeric proteins containing defined domains of Nedd9 and p130Cas/BCAR1 indicate that differences in their substrate domains are responsible for the differential effect on anti-oestrogen resistance [49]. As the substrate domains of these two Cas proteins differ in number, spacing and sequence context of tyrosine motifs, a distinctive pattern of p130Cas/BCAR1 SD tyrosine phosphorylation may be responsible for the recruitment of specific SH2 domain-containing proteins and the subsequent assembly of molecular complexes driving anti-oestrogen-resistant proliferation.

**Figure 4:**
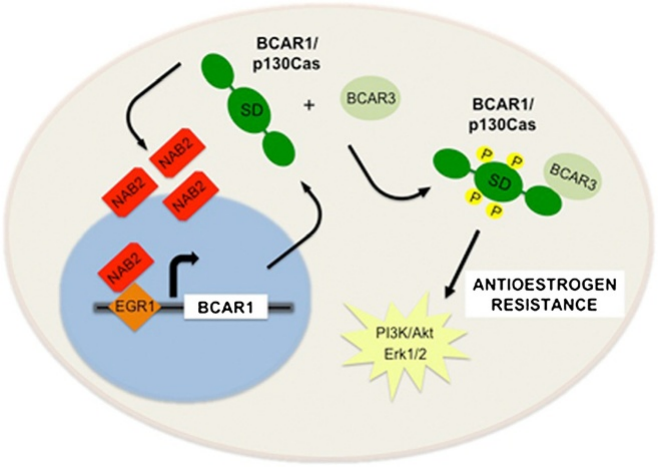
**Schematic representation of the mechanisms underlying p130Cas/BCAR1 up-regulation in tamoxifen-resistant cells and major signalling events in p130Cas/BCAR-dependent anti-oestrogen resistance.** PI3K, phosphoinositide 3-kinase; SD, substrate domain.

An additional mechanism implicated in tamoxifen resistance relies on the interaction between p130Cas/BCAR1 and the SH2-containing protein AND-34/BCAR3. Although there is a clear correlation between the emergence of tamoxifen resistance in breast cancer and the association of BCAR3 with p130Cas/BCAR1, the available data on the functional meaning of this interaction appear controversial [50]. However, it has been recently demonstrated that the binding of BCAR3 to p130Cas/BCAR1 increases the levels of phosphorylated p130Cas/BCAR1, ultimately potentiating Erk1/2 phosphorylation and sustaining BCAR1-dependent anti-oestrogen resistance [51] (Figure 4).

## Conclusion

p130Cas/BCAR1 and Nedd9, two members of the Cas protein family, play a key role at multiple steps of mammary tumourigenesis. Abnormal expression and/or phosphorylation of these two scaffold proteins leads to the disruption of regulatory signalling circuits controlling mammary cell survival, differentiation, proliferation and migration.

Noteworthy, in spite of their structural similarity, p130Cas/BCAR1 and Nedd9 exert both redundant and specific functions in breast cancer cells. Although the functional role of these two proteins has been extensively studied, further investigations are required to fully understand the molecular mechanisms underlying the deregulation of their expression in cancer. Moreover, high-resolution mapping of the interactions between Cas proteins and their pro-growth/pro-migratory binding partners may provide a rationale for the design of novel targeted therapies. Nevertheless, the use of Cas proteins as biomarkers for cancer prognosis and drug responsiveness may improve the clinical management of breast cancer.

## Authors’ original submitted files for images

Below are the links to the authors’ original submitted files for images. Authors’ original file for figure 1 Authors’ original file for figure 2 Authors’ original file for figure 3 Authors’ original file for figure 4

## Abbreviations

ER: Oestrogen receptor
FAK: Focal adhesion kinase
MAPK: Mitogen-activated protein kinase
PI3K: Phosphoinositide 3-kinase
SD: Substrate domain
TGF: Transforming growth factor
TNBC: Triple-negative breast cancer
