# Supplementary figures and images for: Cas proteins: dodgy scaffolding in breast cancer
Source: Breast Cancer Res. 2014 Sep 25;16:443. doi: 10.1186/s13058-014-0443-5 (PMC4384296; doi:10.1186/s13058-014-0443-5)

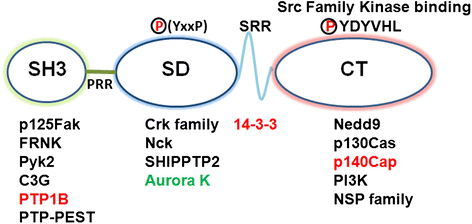

Supplement: Supplementary file 1 — Authors’ original file for figure 1 [file 13058_2014_443_MOESM1_ESM.gif]

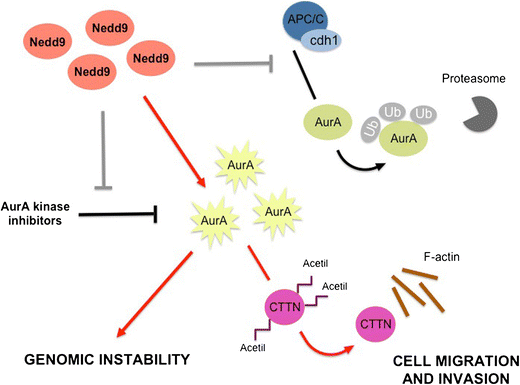

Supplement: Supplementary file 2 — Authors’ original file for figure 2 [file 13058_2014_443_MOESM2_ESM.gif]

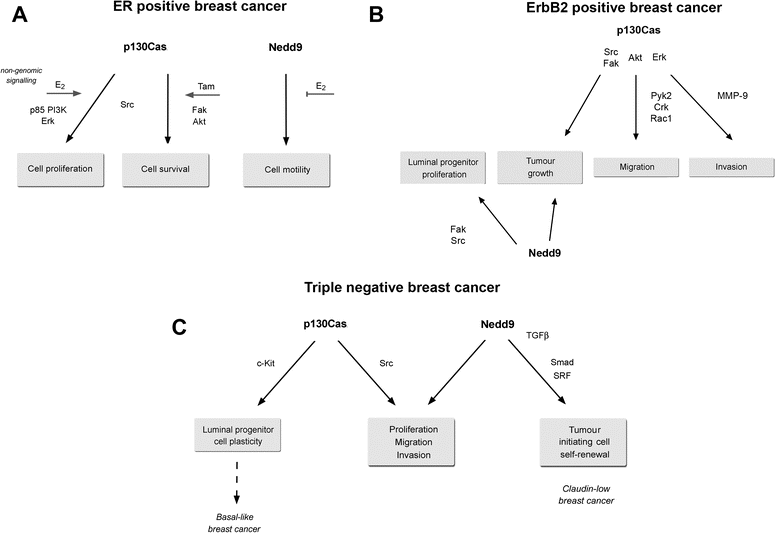

Supplement: Supplementary file 3 — Authors’ original file for figure 3 [file 13058_2014_443_MOESM3_ESM.gif]

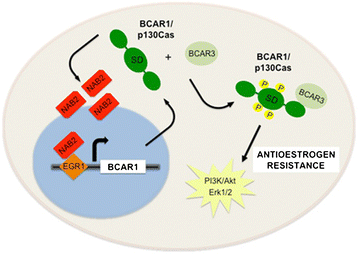

Supplement: Supplementary file 4 — Authors’ original file for figure 4 [file 13058_2014_443_MOESM4_ESM.gif]
